# Altered brain functional connectivity in patients with resistance to thyroid hormone ß

**DOI:** 10.1371/journal.pone.0306538

**Published:** 2024-08-22

**Authors:** Martin Göttlich, Krishna Chatterjee, Carla Moran, Marcus Heldmann, Berenike Rogge, Anna Cirkel, Georg Brabant, Thomas F. Münte

**Affiliations:** 1 Institute of Medical Psychology, University of Lübeck, Lübeck, Germany; 2 Center of Brain, Behavior and Metabolism, University of Lübeck, Lübeck, Germany; 3 Wellcome-MRC Institute of Metabolic Science, University of Cambridge, Cambridge, United Kingdom; 4 Department of Neurology, University of Lübeck, Lübeck, Germany; 5 Department of Psychology II, University of Lübeck, Lübeck, Germany; 6 Department of Internal Medicine I, University of Lübeck, Lübeck, Germany; University of Padova: Universita degli Studi di Padova, ITALY

## Abstract

To investigate changes in brain network organization and possible neurobehavioral similarities to attention-deficit hyperactivity disorder (ADHD), we measured changes in brain resting-state functional connectivity (rs-fMRI) and cognitive domains in patients with resistance to thyroid hormone β (RTHβ) and compared them with those in healthy control subjects. In this prospective case-control study, twenty-one participants with genetically confirmed RTHβ were matched with 21 healthy controls. The Adult ADHD Self-Report Scale (ASRS-v1.1) and ADHD Rating Scale-IV were used to assess self-reported symptoms of ADHD. A voxel-wise and atlas-based approach was used to identify changes in the brain networks. The RTHβ group reported behavioral symptoms similar to those of ADHD. We found evidence of weaker network integration of the lingual and fusiform gyri in the RTHβ group, which was mainly driven by weaker connectivity to the bilateral insula and supplementary motor cortex. Functional connectivity between regions of the default mode network (angular gyrus/middle temporal gyrus) and regions of the cognitive control network (bilateral middle frontal gyrus) was increased in RTHβ patients compared to healthy controls. Increased connectivity between regions of the default mode network and the dorsolateral prefrontal cortex is frequently reported in ADHD and is interpreted to be associated with deficits in attention. Our finding of weaker connectivity of the lingual gyrus to the bilateral insula (salience network) in RTHβ patients has also been reported previously in ADHD and may reflect decreased habituation to visual stimuli and increased distractibility. Overall, our observations support the notion of neuropsychological similarities between RTHβ and ADHD.

## Introduction

Thyroid hormones (TH) play a major role both during brain development and in maintaining the function of the adult brain (Bauer et al., 2008). Both hypo- and hyperthyroidism lead to changes in brain function and result in cognitive alterations. Depending on the degree of hypothyroidism, cognitive effects may range from mild impairments of memory and attention to outright dementia [1]. On the other hand, hyperthyroidism has been associated with inattention and hyperarousal, amongst other cognitive deficits [1,2]. These cognitive changes are underpinned by alterations in brain functions that can be captured by structural and functional brain imaging methods [3–6].

One possible cause of elevated TH levels in the blood is a genetically determined TH receptor dysregulation. Here, we have studied resistance to thyroid hormone β (RTHβ), a rare disorder caused by mutations in the thyroid hormone receptor beta gene (*THRB*) encoding receptor isoforms TRβ2 and TRβ1 [7–11]. A separate gene (*THRA*) encodes thyroid receptor alpha. Receptor isoforms (TRβ2, TRβ1, TRα1) generated from the *THRA* and *THRB* genes exhibit differing tissue distribution [12–14]. The pituitary hormone TSH (thyroid stimulating hormone) stimulates the thyroid gland to produce thyroxine (T4; prohormone), and then triiodothyronine (T3). In turn, thyroid hormones (T3 and T4) in the blood regulate the pituitary release of TSH within the hypothalamic-pituitary thyroid axis mediated by the receptor isoform TRβ2. This negative feedback loop, which stabilizes the level of TH in the blood, is disturbed in RTHβ. This leads to elevated TH and non-suppressed, i.e. normal, TSH levels. TRβ1 is expressed predominantly in liver and kidney, accounting for hepatic resistance to hormone action in RTHβ patients [7]. TRα1 is expressed at the highest levels in the brain, raising the possibility that symptoms similar to attention deficit hyperactivity disorder (ADHD) [15–18] seen in ~50% of RTHβ patients [7] reflect effects of elevated TH on normal TRα1 expressed in the brain.

Attentional control and allocation of mental resources is supported by well characterized brain networks which can be studied by assessing the intrinsic connectivity of the brain using resting state functional magnetic resonance imaging (rs-fMRI). Many studies on ADHD patients have focused on the default-mode network (DMN), which is deactivated during tasks and shows a high level of activity during rest [19], but have also found alterations in other attention-related networks such as the dorsal and ventral attention networks, and cognitive control networks. Adult ADHD patients showed reduced functional connectivity within the DMN compared to normal controls [20,21] which might lead to attentional interference and response variability in ADHD [22]. Other findings were changes in connectivity between the dorsal anterior cingulate and the DMN [20,23] as well as between the dorsolateral prefrontal cortex and the DMN [24]. Decreased functional connectivity within the dorsal and ventral attention networks and increased functional connectivity within right lateralized cognitive control networks and DMN has also been reported [25].

Here, we pursued a network-theoretical approach to evaluate differences in resting-state functional connectivity between RTHβ patients and healthy controls and discuss similarities to changes reported in ADHD. Basically, a network consists of nodes and connections between them. In the analysis of resting fMRI data, these connections are characterized by the strength of the correlation between BOLD time series. We used two approaches, which differ in how the network nodes were defined. In the first case, the voxels of an image formed the network nodes, and in the second case, we used regions defined in a brain atlas as network nodes. Both methods complement each other. In the case of the voxel-based approach, the results are independent of the arbitrary choice of a brain atlas, but due to the high number of connections between the network nodes, we do not study individual connections between voxels, but the number of connections of each voxel to all other voxels in the brain. This metric is referred to as degree centrality in graph theory and its application in neuroscience is well established [26]. Furthermore, the voxel-based approach is sensitive to spatially small effects and to differences in functional connectivity within brain regions, as defined in a selected brain atlas. The atlas-based approach, however, allowed us to study individual connections between network nodes and to correlate them to clinical parameters. We recently used the same method to analyze the effects of experimentally induced thyrotoxicosis [5] and induced subclinical hypothyroidism [27].

As a behavioural phenotype similar to attention deficit hyperactivity disorder (ADHD) has been associated with RTHβ, we looked for aberrant functional connectivity in brain regions mediating attention and cognitive control, i.e., the DLPFC and parietal cortex, in patients. As the disorder is also known to be associated with audio-visual abnormalities, we also hypothesized altered connectivity in the visual, auditory, and sensorimotor network.

## Materials and methods

### Ethics statement

All procedures were approved by the ethical committee of the University of Lübeck. The study was performed in agreement with the Declaration of Helsinki. All subjects gave their written informed consent prior to participation.

### Subjects

In this prospective case-control study, twenty-one participants with TRβ-mutations, all from the UK (mean age 39 y, SD 15.0, 12 women) were matched (age, gender, and education) with 21 healthy controls (mean age 38 y, SD 14, 12 women, all from Lübeck, Germany). Regarding the educational attainment of participants, 8 completed schooling at O-level and 13 at A-level. Moreover, the after-school career was used for matching between groups. All participants were investigated at the University Medical Center Schleswig-Holstein, Campus Lübeck. Subjects were recruited and measured between September 2017 and May 2018. Only authors involved in the recruitment of subjects and who supervised the measurements were aware of the identity of the subjects. The sample size was mainly determined by the availability of subjects with this very rare mutation within a meaningful time frame for the study. The mutations (all heterozygous) in the patients were as follows: R320H (n5), R438H (n4), R429Q (n3), R383C (n2), M310V (n1), G345C (n1), P453S (n1), R243W (n1), T227I (n1), R338W (n1), E460K (n1).

All participants were screened by an endocrinologist prior to the study for general health and had normal structural brain images as evaluated by a neuroradiologist. Laboratory analyses of TSH (thyroid-stimulating hormone), fT3 (free triiodothyronine) and fT4 (free thyroxine) were performed using a one-step immunoassay (Siemens ADVIA Centaur XP; normal ranges: fT3 3.5–6.5 pmol/l, fT4 10–19.8 pmol/l and TSH 0.35–5.5 mU/l). All RTHß patients exhibited elevated fT4 and fT3 with normal TSH levels. This is the expected biochemical profile as normal negative feedback regulation of TSH by thyroid hormones is perturbed in RTHß patients, resulting in raised circulating free thyroid hormones (fT4, fT3) with non-suppressed TSH concentrations. None of the control group had thyroid hormone levels outside the reference range.

A comprehensive psychological battery assessing different cognitive domains was applied. Here, we only consider the two scales related to ADHD symptoms as we hypothesized to find changes in brain network organization similar to ADHD. The Adult ADHD Self-Report Scale (ASRS-v1.1) and the ADHD Rating Scale IV were used to assess self-reported symptoms of ADHD. The ADHD rating scale IV comprises two subscales, inattention and hyperactivity-impulsivity, and is based on 18 items in a 4 point Likert scale format [28]. The Beck Depression Inventory II was used to assess self-reported symptoms typically occurring in depressive disorders [29].

For two participants with TRβ-mutations and two healthy control subjects no resting-state data were acquired. Two patients and one healthy control subject had to be excluded due to excessive head motion during the resting-state recordings. In total, 17 TRβ-mutation carriers and 18 healthy control subjects were included in the analysis. The relevant demographical and clinical information is summarized in Table 1.

**Table 1:** Demographic and psychometric characteristics of RTHβ patients and healthy controls.

	RTHβ mean (SD)	Control mean (SD)	p-value
Gender female/male	9/8	10/8	0.8767^a^
TSH [mU/L]	1.73 (0.77)	2.2 (1.7)	0.3329^b^
fT3 [pmol/L]	8.9 (1.6)	5.14 (0.5)	**0.0000** ^b^
fT4 [pmol/L]	28.5 (5.8)	14.7 (1.7)	**0.0000** ^b^
Age [years]	36.5 (13.3)	35.2 (14.0)	0.6207^b^
ADHD Self-Report Scale (ASRS-v1.1)	96.82 (42.15)	64.17 (23.21)	**0.0094** ^b^
ADHD Rating Scale IV	35.82 (10.58)	26.11 (7.65)	**0.0043** ^b^
BDI	9.24 (7.42)	7.83 (6.67)	0.5616^b^

Significant between-group differences are denoted in bold face.

a) According to a chi^2^-test.

b) Two-sample two-tailed t-test applied.

### MRI acquisition and processing

Structural and functional MR imaging was performed at the CBBM Core Facility Magnetic Resonance Imaging using a 3-T Siemens Magnetom Skyra scanner equipped with a 64-channel head-coil. Functional images were acquired applying a single-shot gradient-recalled echo-planar imaging (GRE-EPI) sequence sensitive to blood oxygen level dependent (BOLD) contrast (TR = 1460 ms; TE = 27 ms; flip angle = 70°; in-plane resolution 3×3 mm2; 192×192 mm^2^ field of view; 50 transversal slices; 3 mm slice thickness; GRAPPA factor 2 and simultaneous multi-slice factor 2; 330 volumes were recorded). Additionally, structural images of the whole brain using a 3D T1-weighted MPRAGE sequence were acquired (TR = 1900 ms; TE = 2.44 ms; TI = 900 ms; flip angle 9°; 1×1×1 mm^3^ resolution; 192×256×256 mm^3^ field of view; acquisition time 4.5 minutes).

The functional data were recorded during an eight-minute resting-state fMRI session. The subjects were instructed to look at a fixation cross and not to move during the measurement.

The functional data was pre-processed using the DPARSF (Data Processing Assistant for Resting-State fMRI) toolbox [30]. The first ten images of each series were deleted, a slice time correction was applied, and a head motion correction was performed by registering all images to the first frame of the series using a six-parameter rigid-body transformation. The functional data were then registered to the SPM12 standard MNI (Montreal Neurological Institute) template (Statistical Parametric Mapping; www.fil.ion.ucl.ac.uk/spm/). Confounding contributions to the data were treated by a nuisance regression including the six head motion parameters, white matter, and ventricular signals. The data were then spatially smoothed with a 6 mm full width half maximum Gaussian kernel and a temporal bandpass filter was applied to all voxel time series (0.01 Hz < f < 0.08 Hz). Volumes with a framewise displacement [31] of more than 0.5 mm (volume to volume) were excluded from the time series along with the previous and the two trailing volumes. One control subject and two subjects from the RTHß group had to be excluded due to strong head motion, i.e., translations larger than 2.5 mm and head rotation larger than 2.5°.

### Degree centrality and regional homogeneity

After pre-processing, time series were available for all voxels in the brain. These time series reflect the temporal development of the signal, i.e., the brain activity, during the resting-state measurement. The degree centrality of a voxel (network node) is defined as the number of connections to all other voxels in the brain (i.e., the network). Two voxels were defined to be connected if their time series were significantly correlated requiring a correlation coefficient larger than ρ = 0.25 (zero-lag Pearson’s linear correlation coefficient). This threshold was imposed to suppress random correlations. For each subject a brain map was obtained reflecting the degree centrality for each voxel. The maps were z-transformed.

Differences in degree centrality between the two experimental groups were investigated applying a two-sample t-test including age and gender as covariates (voxel-wise). Statistical images were assessed for cluster-wise significance using a cluster-defining threshold of p = 0.001. A topological false discovery rate (FDR) procedure was applied to correct for multiple comparisons [32]. The 0.05 FDR-corrected critical cluster size was k = 37. The statistical analysis of between-group effects was performed using SPM12 (http://www.fil.ion.ucl.ac.uk/spm).

For each subject, we extracted the mean degree centrality (z-transformed) in the clusters showing significant between-group differences and performed a regression to remove variance explained by age and gender. We then correlated (Spearman’s rank correlation) the cluster mean z-degree centrality to the ADHD score to investigate whether the changes in z-degree centrality were related to symptom severity.

Voxel-wise regional homogeneity maps for each subject were calculated to assess whether the observed differences in degree centrality were due to regional and short-range changes in functional connectivity in contrast to connectivity changes between distant brain regions in a distributed brain network. For each voxel the Kendall’s coefficient of concordance with its nearest neighbors (n = 24 voxels) was calculated and divided by the mean whole-brain regional homogeneity. The mean regional homogeneity in each cluster where significant differences in degree centrality were found was extracted and compared between the two groups applying a two-sample t-test.

### Atlas based network analysis

We performed a network analysis based on a parcellation of the brain into 392 regions [33]. The time courses of all voxels within a particular brain region were averaged. The 392 mean time courses were then correlated with each other resulting in a symmetric network matrix. Correlations were computed using the Pearson product moment formula. A Fisher z-transformation was applied to all correlation maps prior to the statistical analysis. A two-sample t-test was carried out to identify differences in network connections (i.e., network edges). The ROI-based network analysis was conducted using Matlab® and in-house scripts. Significant differences in functional connectivity between nodes are identified using network-based statistic, NBS [34]. The NBS method is a nonparametric statistical approach to control for the family-wise error rate (FWER) when performing a mass univariate hypothesis testing on all connections within a network. FWER-corrected p-values are calculated for network components rather than individual network edges. We applied a primary test-statistic threshold corresponding to a p-value of p = 0.0001 (two-sided; two sample t-test). The size of a network component was measured by the total number of connections, i.e., the extent of the component. The data is presented in form of chord diagram and the visualization was done using CIRCOS [35].

The statistical analysis of demographic, clinical and behavioural data was performed using Matlab®. If not stated otherwise we report the mean and standard deviation of the data.

We used the Automatic Anatomic Labelling (AAL) brain atlas to localize or identify neuroanatomy [36].

MRIcroGL (https://www.nitrc.org/projects/mricrogl/), among others, was used to create the figures.

## Results

Behavioral and clinical data are summarized in Table 1. RTHβ patients reported significantly higher ADHD symptoms according to both scales: the Adult ADHD Self-Report Scale (ASRS-v1.1) and the ADHD Rating Scale IV. The BDI score was not significantly different. All RTHβ patients exhibited elevated fT4 and fT3 with normal TSH levels (normal ranges: fT3 3.5–6.5 pmol/l, fT4 10–19.8 pmol/l and TSH 0.35–5.5 mU/l).

**Fig 1:**
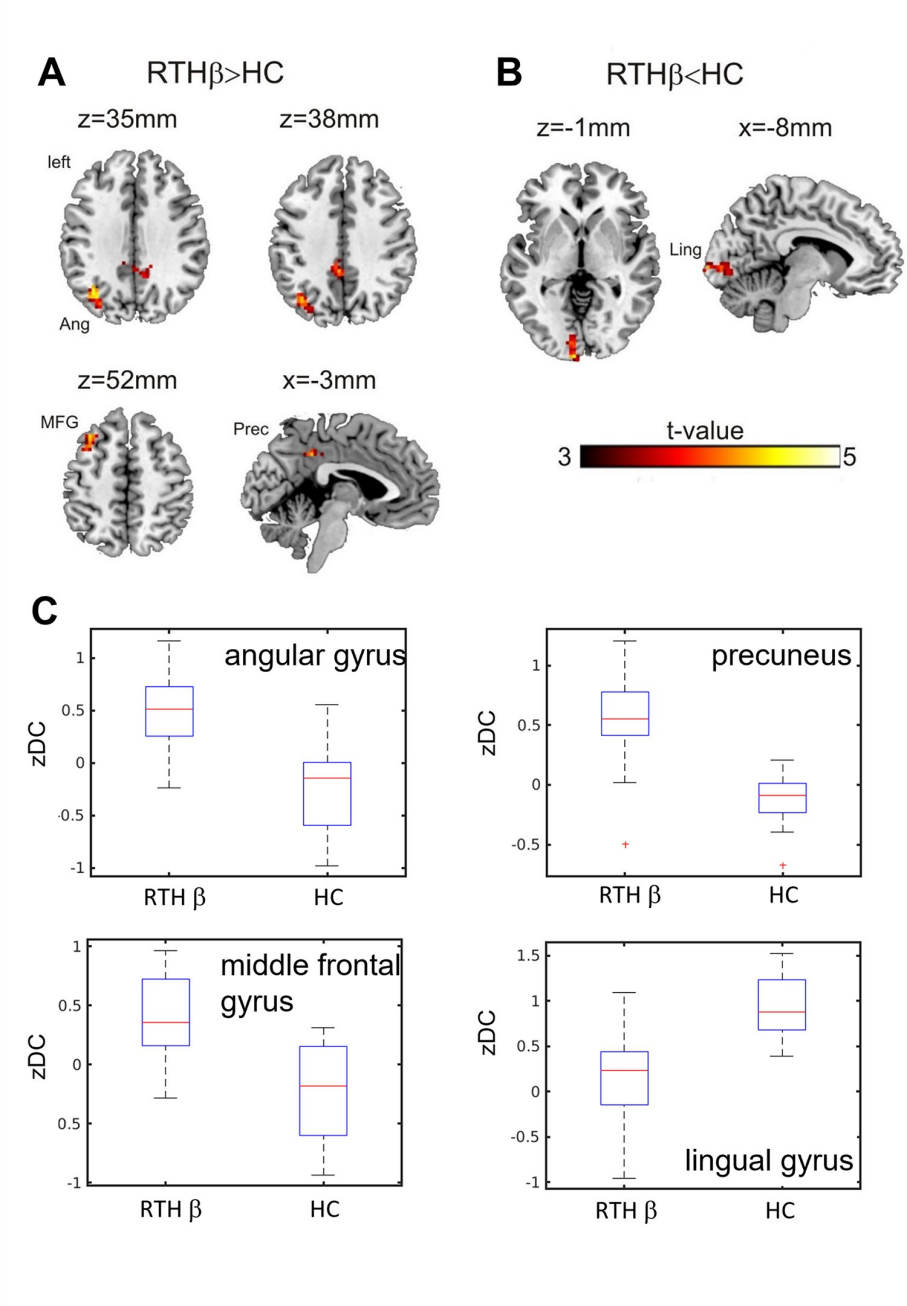
Degree centrality. A) Increased z-degree centrality in the RTHβ group compared to healthy controls (topological FDR correction p<0.05). B) The RTHβ group showed decreased z-degree centrality in the lingual gyrus. C) Boxplots describing the distribution of the z-degree centrality in the angular gyrus, precuneus, middle frontal gyrus and lingual gyrus clusters for the RTHβ group and for healthy controls. Copyright (C) 1993–2004 Louis Collins, McConnell Brain Imaging Centre, Montreal Neurological Institute, McGill University.

The RTHβ group showed a significantly higher (topological FDR correction; q<0.05) voxel-wise degree centrality in the left angular gyrus, the precuneus (cluster extending to the posterior cingulate cortex) and the left middle frontal gyrus compared to healthy controls (Fig 1A and Table 2). The degree centrality was significantly decreased in the left lingual gyrus (Fig 1B and Table 2). The adjusted cluster level probability, the number of voxels per cluster (k) and the peak T-scores of local maxima and their MNI coordinates are listed in Table 2. The distribution of the mean degree centrality for each of the four clusters and for the RTHβ group and healthy controls separately is depicted in Fig 1C.

**Table 2:** Significant between-group differences in degree centrality comparing the RTHβ group to healthy controls.

Anatomical region		q (FDR) (cluster)	k	local maximum (x, y, z) [mm]			T_33_ (peak)
a) RTHβ>HC							
Precuneus, PCC		0.016	45	15	-51	33	4.85
				-3	-42	39	4.29
				3	-30	42	3.71
Middle frontal gyrus	L	0.025	37	-33	24	54	4.65
				-33	15	51	4.13
Angular gyrus Middle occipital gyrus	L L	0.012	55	-36	-66	36	4.57
				-30	-72	33	4.15
b) RTHβ<HC							
Lingual gyrus	L	0.022	47	-6	-102	0	4.51
				-9	-90	0	4.02
				-9	-84	-6	3.64

Clusters with significant differences in degree centrality (cluster defining threshold p<0.001; topological FDR-correction q<0.05). Anatomical region, adjusted cluster level probability and number of voxels per cluster (k) are listed. The table shows 3 local maxima (Montreal Neurological Institute coordinates) more than 8.0 mm apart and the corresponding peak T-scores. Abbreviations: Posterior cingulate cortex (PCC).

A seed-based functional connectivity analysis using the cluster in the middle frontal gyrus as a seed revealed higher functional connectivity to regions of the default mode network, i.e. the precuneus and the middle temporal gyrus, in RTHβ patients compared to healthy controls (S1 Fig). Please note that this is a purely descriptive analysis, as a seed region was selected that has already shown significantly different functional connectivity and the result is therefore not independent and biased. However, this analysis reveals regions that contribute most to the observed effect.

**Fig 2:**
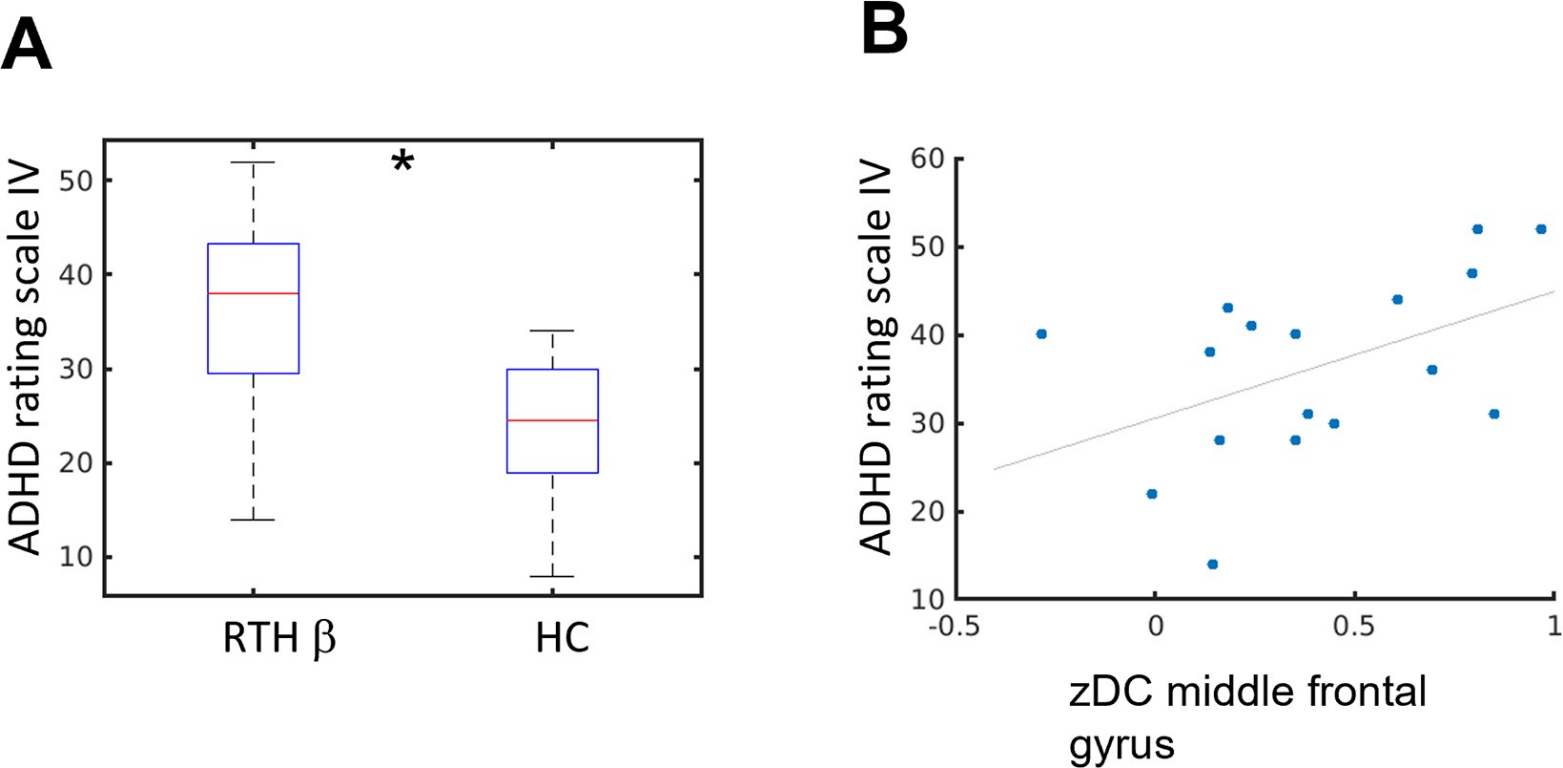
Middle frontal gyrus degree centrality correlates with ADHD score. A) Boxplot describing the distribution of the ADHD rating scale IV for the RTHβ group and for healthy controls. The RTHβ group reported significantly stronger ADHD symptoms compared to healthy controls: 35.82 (SD 10.58) vs. 26.11 (SD 7.65); two-sample t-test p = 0.0043. B) Scatter plot visualizing the relationship between the ADHD rating scale IV and the z-degree centrality in the middle frontal gyrus (MFG) in the RTHβ group. We observed a significant correlation between ADHD symptom strength and MFG z-degree centrality: Spearman’s rho 0.5 and p = 0.0398.

The distribution of the ADHD scores (ADHD Rating Scale IV) in both groups are described in Fig 2A. We observed a significant correlation between ADHD symptom strength and MFG z-degree centrality: Spearman’s rho 0.5 and p = 0.0398 (Fig 2B).

We did not observe between group differences in regional homogeneity (S2 Fig).

**Fig 3:**
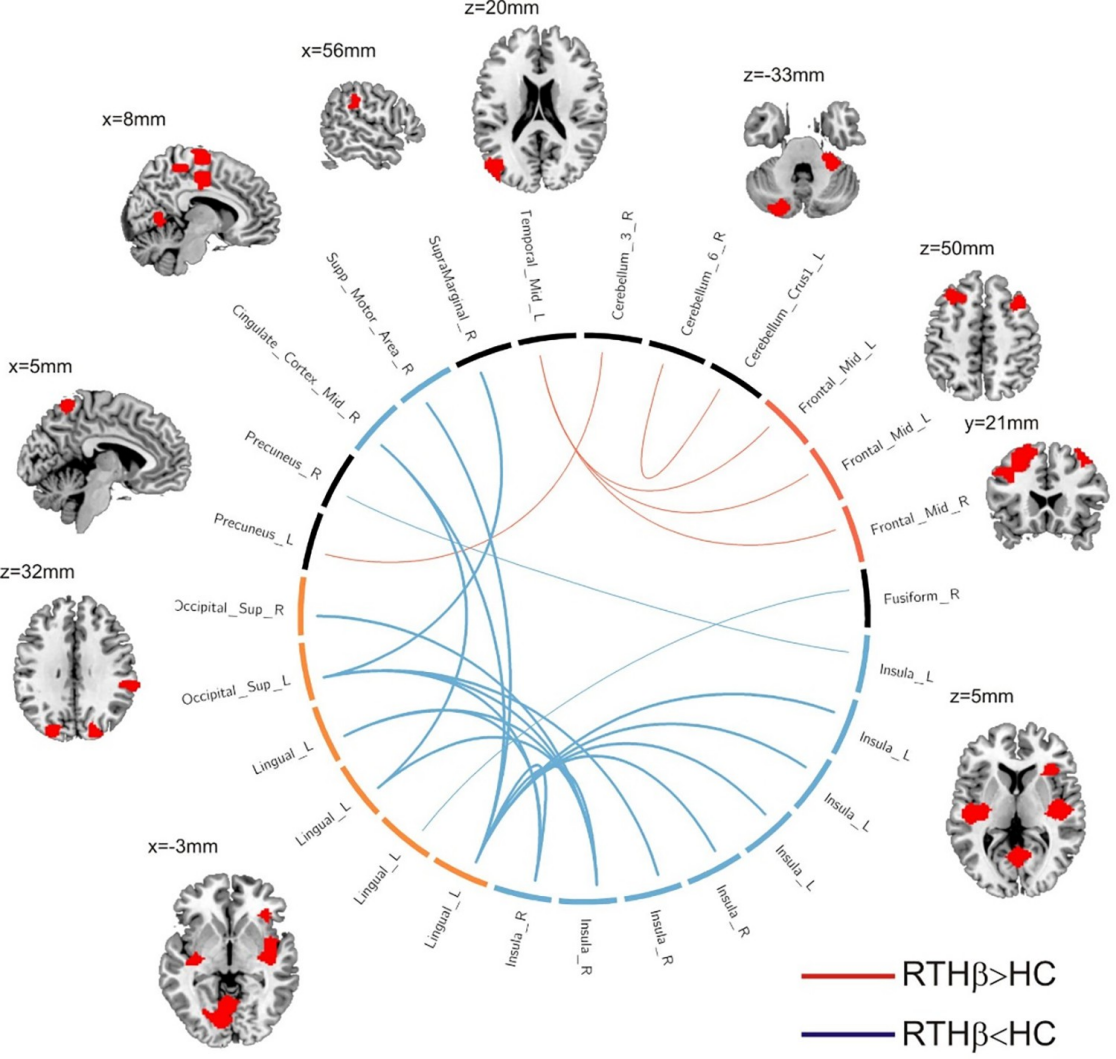
Network analysis. Significant differences in functional connectivity as revealed by the ROI-based network analysis. Red and blue lines indicate increased and decreased connectivity in the RTHβ group, respectively. Shown are results controlling for the family-wise error rate using network–based statistic (corrected p<0.05; broad blue lines indicating significantly stronger connectivity in healthy controls) and uncorrected results (p<0.0001; thin blue and red lines). Copyright (C) 1993–2004 Louis Collins, McConnell Brain Imaging Centre, Montreal Neurological Institute, McGill University.

Correcting for the FWER we identified a network component showing a significantly decreased functional connectivity comparing the RTHβ group to healthy controls (p = 0.014; FWER corrected applying the NBS; Fig 3; broad blue lines). The result indicated a weaker connectivity between the lingual and superior occipital gyri on the one hand and the insula and the supplementary motor area and the cingulate cortex on the other hand. Also shown are uncorrected results applying a rather stringent p-threshold of p<0.0001. As indicated by the thin red lines in Fig 3 the RTHβ group showed a significantly stronger connectivity between the left posterior middle temporal gyrus (as part of the default mode network; S1 Fig) and the bilateral middle frontal gyri. The results are also summarized in Table 3.

**Table 3:** Differences in functional connectivity between network nodes.

Node 1 (MNI coordinates in mm)	Node 2 (MNI coordinates in mm)	T_33_
a) RTHβ<HC		
Insula (left) (-34,11,-8)	Precuneus (right) (3,-39,58)	4.59
Insula (right) (38,-22,14)	Lingual gyrus (left) (-25,-70,-13)	4.94
Supramarginal gyrus (right) (61,-35,34)	Superior occipital gyrus (left) (-23,-84,31)	4.52
Insula (left) (-36,-17,1)	Lingual gyrus (left) (-25,-70,-13)	4.94
Insula (left) (-39,-14,15)	Lingual gyrus (left) (-25,-70,-13)	4.44
Insula (right) (42,2,7)	Superior occipital gyrus (left) (-23,-84,31	4.91
Fusiform (right) (39,-15,-25)	Lingual gyrus (left) (-31,-85,-15)	4.76
Insula (right) (42,-0,-10)	Superior occipital gyrus (right) (21,-82,37)	4.46
Insula (right) (42,-0,-10)	Lingual gyrus (left) (-25,-70,-13)	4.89
Insula (right) (42,-0,-10)	Superior occipital gyrus (left) (-23,-84,31)	5.33
Insula (right) (42,-0,-10)	Lingual gyrus (left) (-11,-77,-10)	4.66
Insula (right) (42,-0,-10)	Lingual gyrus gyrus (0,-63,1)	4.89
Lingual gyrus (left) (-25,-70,-13)	Insula (right) (41,-14,-1)	5.52
Lingual gyrus (left) (-25,-70,-13)	Superior temporal gyrus (left) (-48,-20,8)	4.58
Lingual gyrus (left) (-25,-70,-13)	Cingulate cortex (right) (8,-14,45)	4.50
Lingual gyrus (left) (-25,-70,-13)	Supp. motor area (right) (8,-15,70)	4.64
Insula (right) (41,-14,-1)	Superior occipital gyrus (left) (-23,-84,31)	4.78
Insula (right) (41,-14,-1)	Lingual gyrus (0,-63,1)	4.46
Lingual gyrus (left) (-11,-77,-10)	Cingulate Cortex (right) (8,-14,45)	4.64
b) RTHβ>HC		
Middle temporal gyrus (left) (-44,-76,19)	Middle frontal gyrus (right) (34,30,42)	-4.54
Middle temporal gyrus (left) (-44,-76,19)	Middle frontal gyrus (left) (-29,24,45)	-4.93
Middle temporal gyrus (left) (-44,-76,19)	Middle frontal gyrus (left) (-43,23,36)	-4.59
Cerebellum (right) (29,-38,-34)	Cerebellum (left) (-24,-83,-30)	-4.67
Cerebellum (right) (19,-27,-25)	Precuneus (left) (-2,-54,63)	-5.02

Network nodes are labelled according to the AAL atlas [36]. The Montreal Neurological Institute (MNI) coordinates of the ROI center are given in mm for each node. Also listed is the T-value for each connection (ndf 33).

## Discussion

We evaluated changes in brain network organization in patients with TRβ-mutations (RTHβ group) compared to healthy control subjects. The voxel-wise degree centrality served as marker for altered functional connectivity. An atlas-based approach complemented this analysis and allowed us to study the observed alterations in more detail. Network nodes were defined using a brain atlas which subdivides the brain into 392 regions. Connectivity between these brain regions was derived by correlating regional BOLD time courses. We found a good agreement between the atlas- and voxel-based analyses concerning the affected brain regions. Our results from the atlas-based analysis and the absence of differences in regional homogeneity point to functional connectivity changes between distant brain regions and between well-known resting-state brain networks rather than intra-regional and short-range changes.

We found evidence for stronger functional connectivity between regions of the default mode network (angular gyrus/middle temporal gyrus) and regions of the cognitive control network (bilateral middle frontal gyrus) comparing RTHβ patients to healthy controls. This connectivity alteration has been frequently reported in ADHD patients [20,23–25,37]. Posner et al. [38] systematically reviewed resting-state studies comparing ADHD patients with healthy controls and pointed to several studies reporting a stronger connectivity (or decreased inverse-correlation) between the default mode network (DMN) and the cognitive control network (CCN). During demanding tasks requiring attention the DMN is suppressed while CCN activity is increased [19,39,40]. Decreased deactivation of the DMN during a task is associated with momentary lapses in attention [41] and higher error rate in a stop signal task [42]. The authors relate this observation to impaired attention due to interference by DMN activity which might mediate mind-wandering. Posner et al. [38] suggested that an increased DMN-CCN resting-state functional connectivity (or decreased inverse-correlation) might express higher proneness to mind-wandering and thus might be interpreted as neural underpinnings of attention deficits in ADHD. The increased degree centrality in the left middle frontal gyrus was positively correlated to self-reported ADHD symptom severity in the RTHβ group. This supports the notion of a neurophysiological and behavioral phenotype in RTHβ that is indistinguishable from ADHD. In a previous work investigating the same cohort, we analyzed changes in brain structure in subjects with RTHβ [43]. The RTHβ-group showed increased gray matter volume in the right middle frontal gyrus which was positively correlated with the ADHD scores. While we observed in our degree centrality analysis an increased functional connectivity in the left middle frontal gyrus which was positively correlated to the ADHD score, our ROI-based analysis revealed increased connectivity of the bilateral middle frontal gyrus (Fig 3). In our previous work, we [43] also reported increased gray matter volume in the precuneus (cluster peak MNI coordinates -8, -45, 78 mm) and increased cortical thickness in the bilateral parietal cortex (cluster peak MNI coordinates 18, -89, 25 and -27, -90, 32 mm). Interestingly, these regions correspond to brain regions which showed altered functional connectivity in our network analysis (Fig 3 and Table 3: precuneus, left and right superior occipital gyrus). Note, that the brain regions are labelled differently as we used here the ROI center of mass coordinates as reference and in our previous work the coordinates of the peak cluster t-value. However, the correspondence between the structural and functional data needs to be further explored using an independent data set, but there is an interesting correspondence between the results in terms of the regions involved and the correlation with the ADHD scale.

Furthermore, our observation of an increased functional connectivity between the temporal lobe and the CCN is in agreement with our previous study in which we investigated rs-fMRI in experimentally induced thyrotoxicosis [5]. Liu et al. [44] investigated resting-state functional connectivity in untreated patients with hyperthyroidism. As a measure for altered regional cerebral activity in patients compared to healthy controls they used the amplitude of low-frequency fluctuation (ALFF) approach and report evidence for significantly decreased ALFF in the posterior cingulate cortex (MNI coordinates of peak t-value 9, -60, 27 mm) in the patient group. Interestingly, a strong spatial overlap exists with the precuneus/PCC cluster, where we found increased whole-brain functional connectivity (degree centrality; Fig 1 and Table 2). In [45] Liu et al. performed a resting-state fMRI study in patients with hyperthyroidism using seed regions in the salience (SN), default mode (DMN) and executive control network (ECN) networks. One striking observation is the central role of the precuneus/posterior cingulate cortex. Patients show increased functional connectivity to the insula (left and right) and the dorsal anterior cingulate cortex. In this aspect, our data fits in with the literature. We see parallels between the patient cohorts, although there are different causes for the elevated hormone levels and although the RTH patients have a syndrome that has existed since birth. In order to specifically search for possible differences, it would make sense to directly compare RTHβ patients and patients with hyperthyroidism of other etiologies. A seed-based analysis using the precuneus/PCC as a seed appears to be promising here.

The RTHβ group exhibited a weaker connectivity between the visual network (specifically the lingual gyrus and superior occipital cortex) and brain regions comprising the salience resting state network: the bilateral insula, the dorsal cingulate cortex, and the supplementary motor cortex (Fig 3). This is in accordance with Zhao et al. [46] who found evidence for decreased functional connectivity between the insula and the lingual gyrus in ADHD. The salience network is involved in detecting and filtering salient stimuli and in recruiting relevant functional networks [47,48]. During a visual oddball task children and adolescents with ADHD showed increased activation of the bilateral lingual gyrus compared to healthy control subjects [49]. The authors argue that attention deficits might be associated with increased sensitivity to external salient stimuli. This is also supported by the work of Jansiewicz et al. [50] who found evidence for impaired habituation to visual stimuli in children with ADHD. The weaker functional connectivity between the salience network and the lingual gyrus might reflect a weaker disengagement of the lingual gyrus leading to hyperactivity in that brain region which, behaviorally, results in increased distractibility.

We found indications for a weaker functional connectivity between the salience network and the precuneus which is part of the default mode network. The insula is involved in the allocation of salience to stimuli by disengagement of the DMN, processes deemed essential for attention, working memory and higher order cognition [47,51]. Zhao et al. [46] stated that “the altered RSFC may suggest that the anterior insula fails to disengage the DMN” in ADHD resulting in attention and working memory deficits. However, more studies are needed to explicitly test the interpretation of our results. For example, one possible approach would be to investigate the effective connectivity between the insula and the lingual gyrus during a visual oddball task, enabling us to directly test the hypothesis that failed disengagement by the salience network leads to lingual gyrus hyperactivity.

A major limitation of our study is that no ADHD group was included, which would have allowed a direct comparison. This limitation was at least partially addressed by examining the correlation between the behavioral data, i.e., the ADHD scale, and observed alterations in functional connectivity. Furthermore, the resting state fMRI literature regarding ADHD is extensive and our interpretation of the results is therefore based on robust prior findings. However, considering this limitation, the results of our study can only provide initial indications of neurobiological similarities between ADHD and TBR and motivate future, more elaborate studies with an additional experimental group of ADHD patients. Our proposed interpretations of the results can then serve as hypotheses and be specifically tested. Approaches for testing our hypotheses and interpretations have been discussed. It should be emphasized that this is the first resting state fMRI study in RTHβ patients and therefore we see our results as a valuable contribution. The representative composition of the sample and the robustness of the results argue for generalizability of the results, while the relatively small sample size, mainly due to the rarity of this mutation, may limit it.

Our results support the hypothesis of RTHβ resulting in symptoms and related brain network changes typically associated with ADHD via induction of central hyperthyroidism. This converges with evidence reported recently showing an ADHD-like phenotype for performance monitoring [52].

## Conclusion

Behaviorally, the RTHβ patient group showed symptoms similar to those of ADHD. Interestingly, we found evidence for increased connectivity between regions of the default mode network and the dorsolateral prefrontal cortex which is frequently reported for ADHD patients, and which is interpreted to be associated with attention deficits. In addition, our finding of weaker connectivity of the lingual gyrus to the bilateral insula (salience network) in the RTHβ group has also been reported in ADHD and may be related to decreased habituation to visual stimuli and increased distractibility. Our results support the notion of a neurophysiological and behavioral phenotype in RTHβ that is indistinguishable from ADHD.

## Supporting information

S1 Checklist STROBE statement—checklist of items that should be included in reports of observational studies. (DOCX)

S1 Fig Middle frontal gyrus functional connectivity. Shown is the default mode network (from https://www.nitrc.org/projects/rsnatlascxsubcx/) in blue and A) increased middle frontal gyrus functional connectivity in RTHb compared to healthy controls (t>2) in the left middle temporal/angular gyrus and the precuneus and B) the “Temporal Mid L” region shown in Fig 3. This illustrates stronger functional connectivity of the middle frontal gyrus to typical default mode network brain regions as the precuneus and the middle temporal/angular gyrus in RTHb. Copyright (C) 1993–2004 Louis Collins, McConnell Brain Imaging Centre, Montreal Neurological Institute, McGill University.
(TIF)

S2 Fig Regional homogeneity. Regional homogeneity in the four clusters where we observed significant differences in z-degree centrality between the RTHb group and healthy controls. There were no significant between group differences in regional homogeneity neither in the angular gyrus, the precuneus, the middle frontal gyrus nor the lingual gyrus.
(TIF)
