# Supplementary figures and images for: Altered brain functional connectivity in patients with resistance to thyroid hormone ß
Source: PLoS One. 2024 Aug 22;19(8):e0306538. doi: 10.1371/journal.pone.0306538 (PMC11341041; doi:10.1371/journal.pone.0306538)

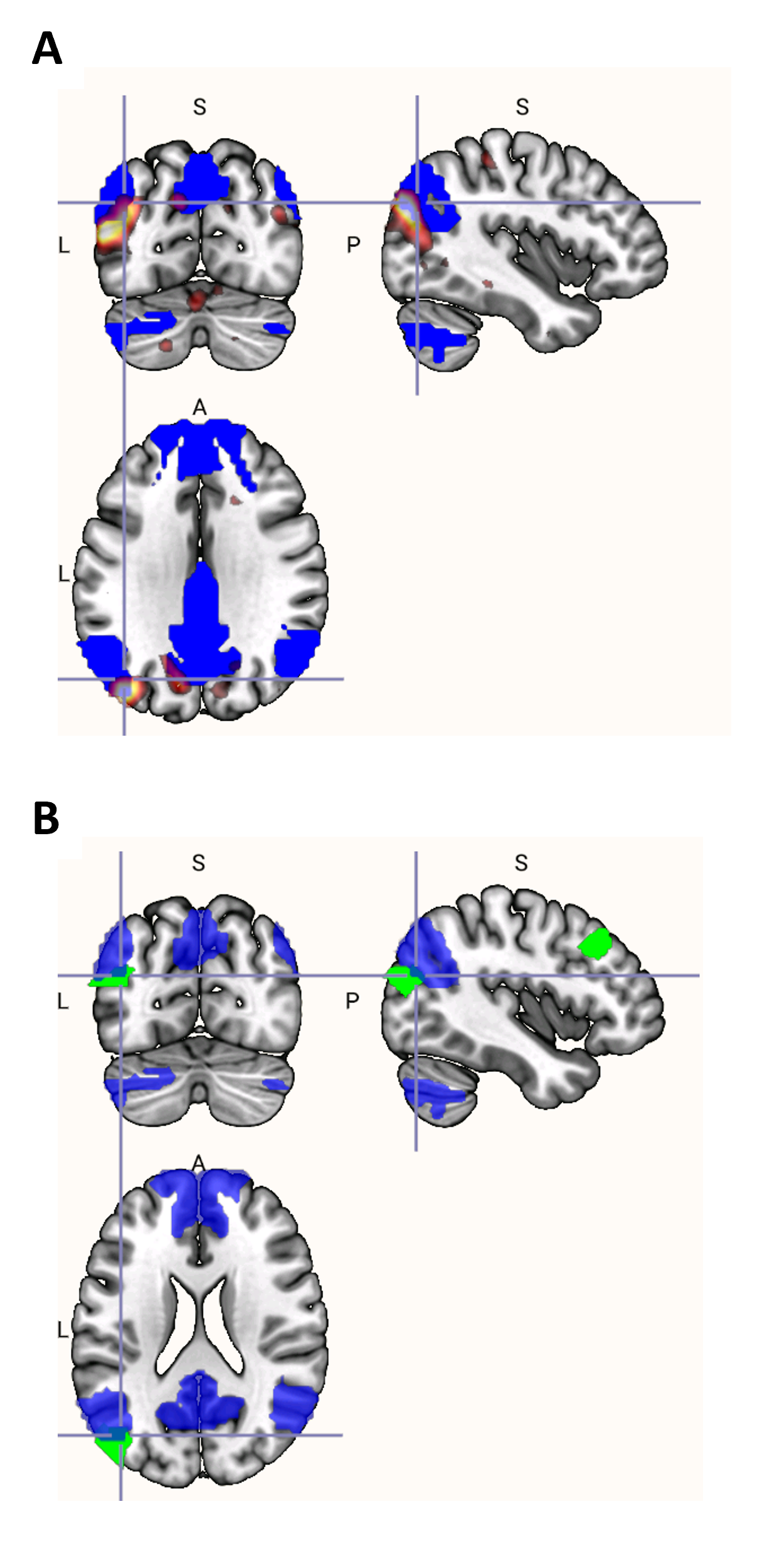

Supplement: S1 Fig — Shown is the default mode network (from https://www.nitrc.org/projects/rsnatlascxsubcx/) in blue and A) increased middle frontal gyrus functional connectivity in RTHb compared to healthy controls (t>2) in the left middle temporal/angular gyrus and the precuneus and B) the “Temporal Mid L” region shown in Fig 3. This illustrates stronger functional connectivity of the middle frontal gyrus to typical default mode network brain regions as the precuneus and the middle temporal/angular gyrus in RTHb. Copyright (C) 1993–2004 Louis Collins, McConnell Brain Imaging Centre, Montreal Neurological Institute, McGill University. (TIF) [file pone.0306538.s002.tif]

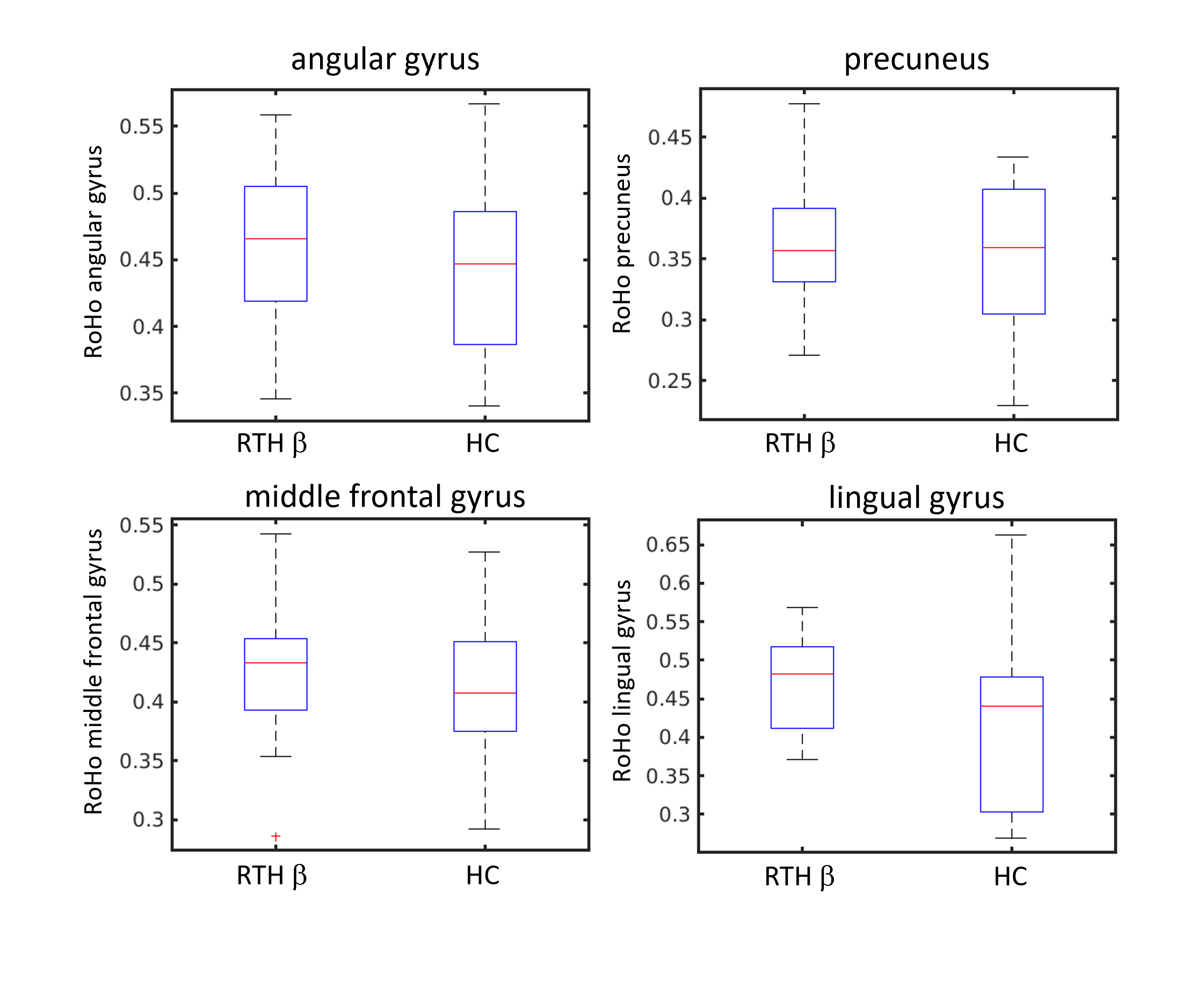

Supplement: S2 Fig — Regional homogeneity in the four clusters where we observed significant differences in z-degree centrality between the RTHb group and healthy controls. There were no significant between group differences in regional homogeneity neither in the angular gyrus, the precuneus, the middle frontal gyrus nor the lingual gyrus. (TIF) [file pone.0306538.s003.tif]
